# Introduction into PPPM as a new paradigm of public health service: an integrative view

**DOI:** 10.1186/1878-5085-3-16

**Published:** 2012-11-09

**Authors:** Tatiana A Bodrova, Dmitry S Kostyushev, Elena N Antonova, Shimon Slavin, Dmitry A Gnatenko, Maria O Bocharova, Michael Legg, Paolo Pozzilli, Mikhail A Paltsev, Sergey V Suchkov

**Affiliations:** 1 National Research University ‘Higher School of Economics’, Moscow, Russia; 2 I.M.Sechenov First Moscow State Medical University, Moscow, Russia; 3 Moscow State Medical Dental University, Moscow, Russia; 4 National Research Centre Kurchatov Institute, Moscow, Russia; 5 Centre for Health Informatics, Sydney, Australia; 6 Department of Endocrinology and Diabetes, Rome University, Rome, Italy; 7 The International Center for Cell Therapy & Cancer Immunotherapy (CTCI), Weizman Center, Israel

**Keywords:** Predictive, preventive, and personalised medicine, Subclinical, Omics, Bioinformatics, Biopredictors, Biomarkers, Ethics, Policy, Legacy, Economy, Integrative medical approach

## Abstract

In the present state of healthcare, usual medical care is generally given to the already diseased person, while the key link—personal health monitoring underlain by predictive, preventive, and personalised medicine (PPPM) techniques that are being intensively elaborated worldwide—is simply missing. It is this link, based on the recognition of *subclinical* conditions, *prediction*, and further *preventive* measures, that is capable of regulating morbidity and diminishing the rates of disability among able-bodied population, thus significantly cutting the traditionally high costs of treating the already diseased people. To achieve the above-mentioned goal—the elaboration of the PPPM concept and its practical implementation—it is necessary to create a fundamentally new strategy based upon the subclinical recognition of the signs—*bioindicators* of cryptic abnormalities long before the disease clinically manifests itself. The implementation of PPPM programme requires an adjusted technology for the proper interpretation of diagnostic data, which would allow for the current ‘physician-patient’ model to be gradually replaced by a novel model, ‘medical advisor-healthy men-at-risk’. This is the reason for an additional need in organising combinatorial scientific, clinical, training and educational projects in the area of PPPM to elicit the content of this new branch of medicine.

## Review

### Introduction

Over the course of its history, medicine has given special attention to the already diseased individual, focusing on studying a type of disorder (*nosology*) rather than one’s health or the so-called *pre-nosological* conditions, the latter being left in the shade. Meanwhile, at present, medicine is undergoing a paradigm shift from the real-time diagnostics and treatment to *prediction* and *prevention*[1-3].

**Figure 1:**
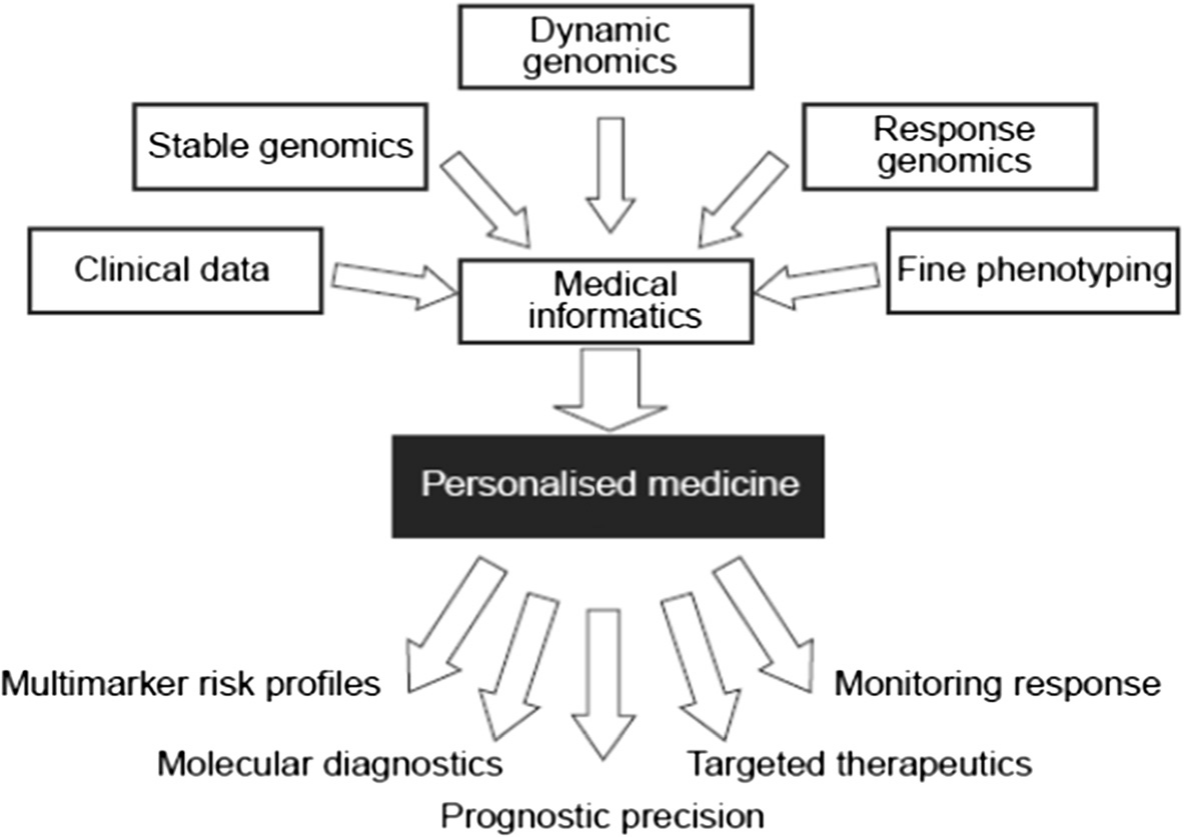
The scheme depicts the underpinnings of personalised medicine and principle approaches to be included into the PPPM protocols.

This major upheaval is expected to transform the nature of healthcare from *reactive* to *preventive*[4]. The changes will be catalysed by a new system approach to disease that will trigger the emergence of *personalised* medicine—a medicine that focuses on the integrated diagnosis, treatment, and prevention of disease in individual patients (Figure 1). This change is rooted in new science [5].

**Figure 2:**
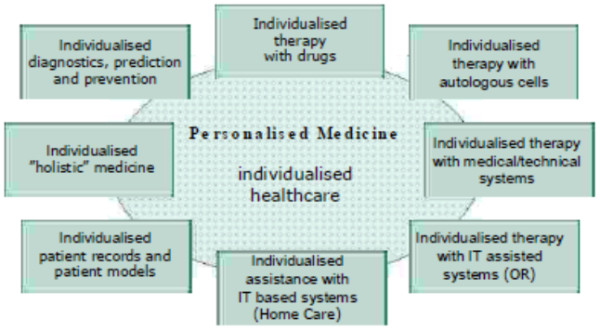
The overarching idea of personalised (individualised) medicine with basic branches aimed at subclinical diagnosis and preventive measures.

The convergence of system approaches to disease, new measurement and visualisation technologies [6,7], and new computational and mathematical tools can be expected to allow our current, largely reactive mode of medicine, where we wait until the patient becomes ill before responding, to be replaced over the next 10 to 20 years with predictive, preventive, and personalised medicine (PPPM) (Figure 2) that will be cost-effective and increasingly focused on the ‘well-being’ concept [2,3,5].

**Figure 3:**
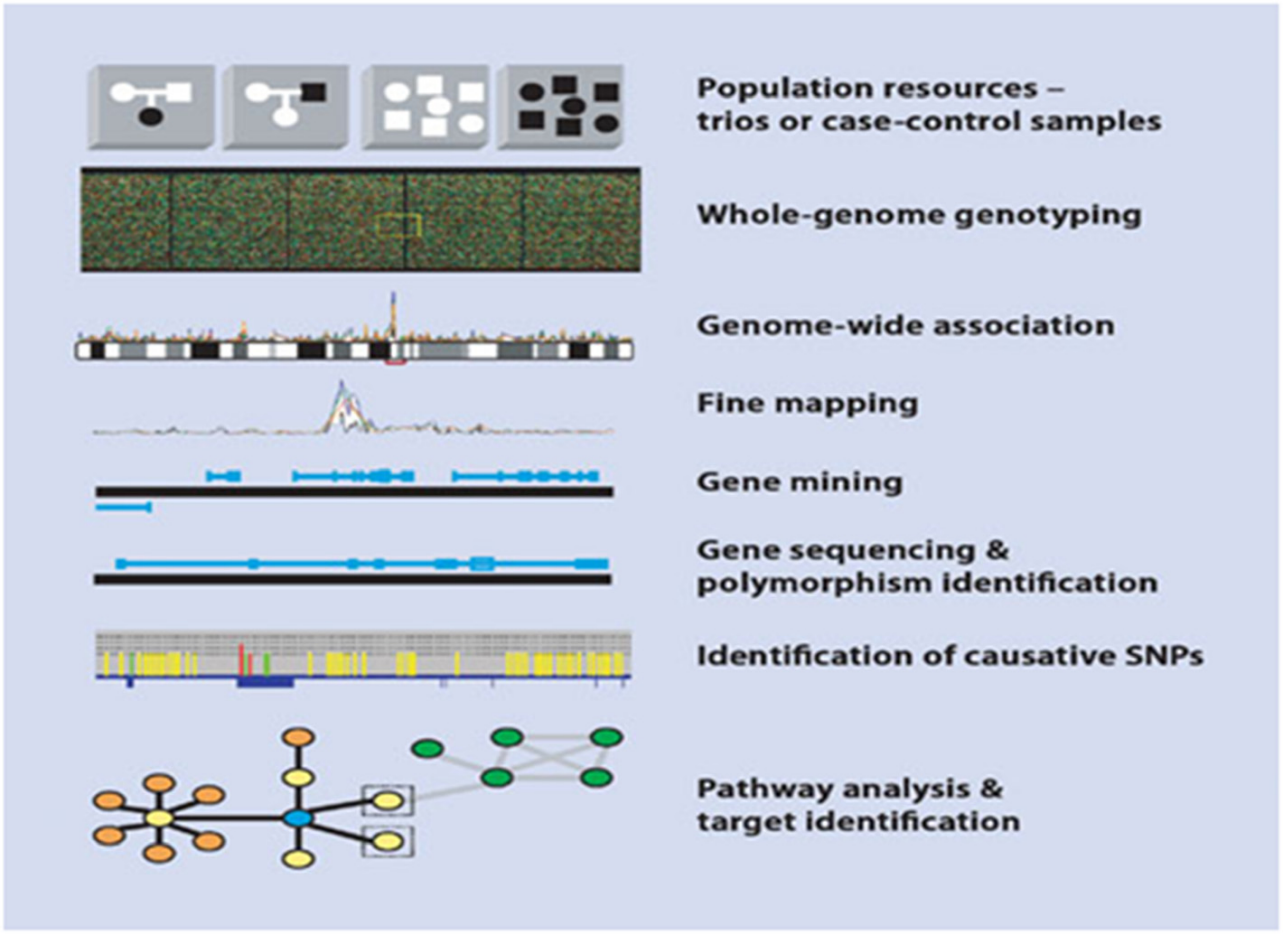
The role of genomics in new target identification and screening of causative mutations.

**Figure 4:**
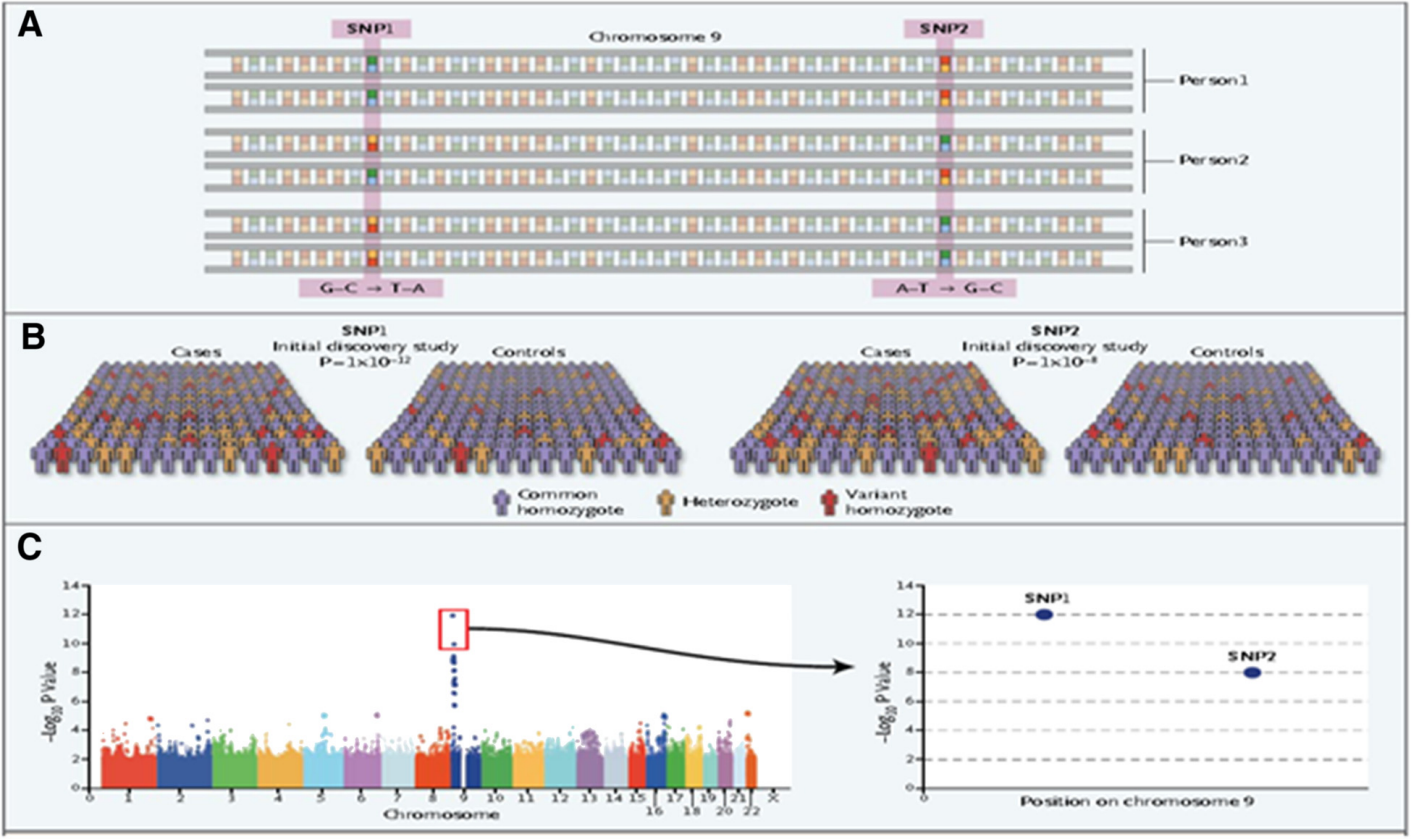
**Bioinformatics as a valuable tool for processing of large data values in indicative research, genome-wide analysis and large-scale population studies.** (**A**) Simultaneous genotyping of more than 500,000–1,000,000 SNPs. (**B**) Initial discovery study with large patient and control sample collection. (**C**) Statistical analysis (probability plot for association with a certain disease) and independent replication of top results.

It was at the turn of the 1990s that this dramatic turning point in the view of the role and place of medicine in healthcare system occurred, following the implementation of the achievements of innovative omics technologies (*genomics*, *transcriptomics*, *proteomics*, *metabolomics*, etc.) (Figure 3) and *bioinformatics* (Figure 4) into clinical medicine which make it possible to penetrate tissues and organs and create conditions to secure the visualisation of lesion foci that is previously unknown to clinicians [8].

### Innovative omics technologies as the fundamental basis of PPPM

Omic-based *presymptomatic prediction* (see Figures 3, and 4) of an illness and allied events, finer diagnostic subclassifications, and improved risk assessment tools applied early in life will permit more-targeted and cost-effective intervention in children and adults [9].

### Genomics

**Figure 5:**
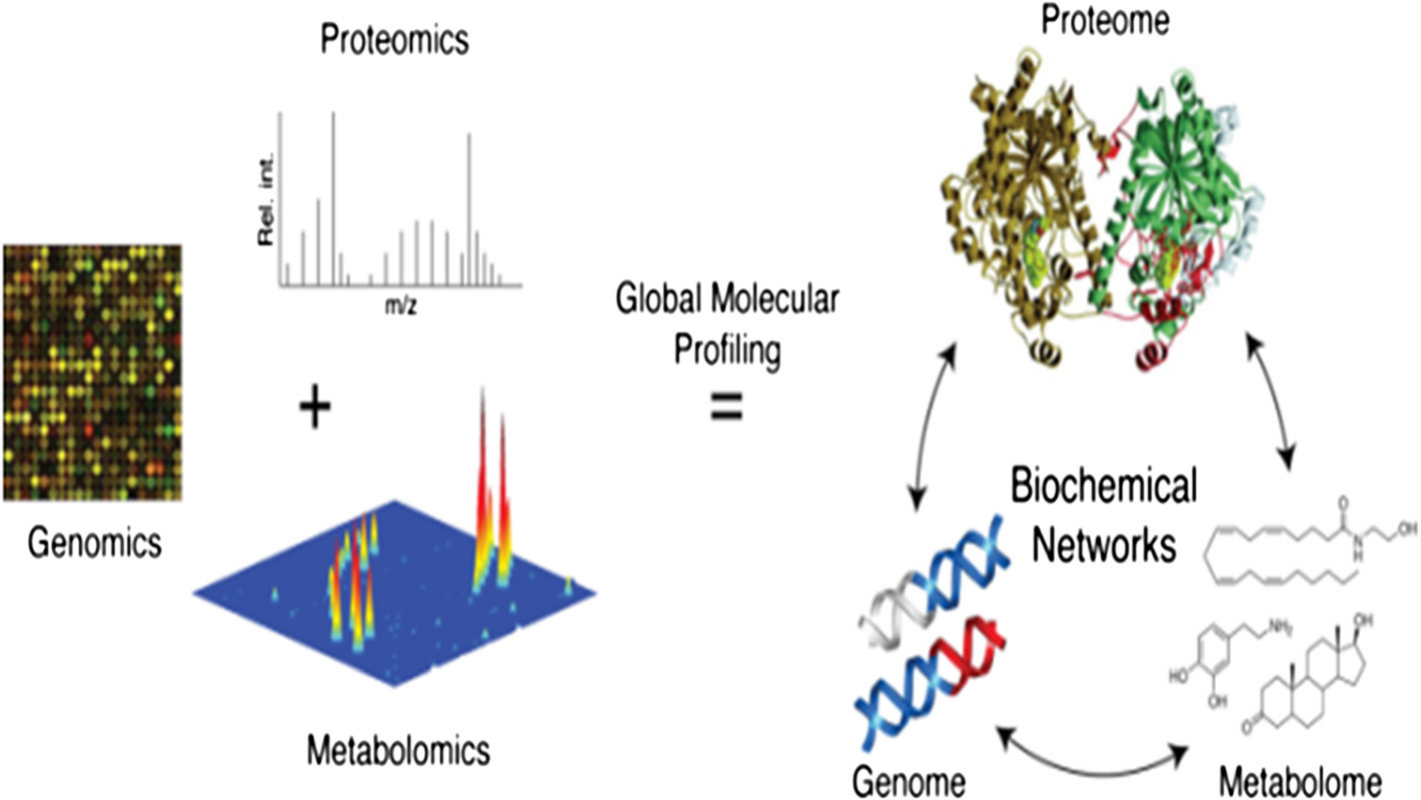
The interrelations between the basis of PPPM (genomics, proteomics, and metabolomics) and their application for global molecular profiling.

**Figure 6:**
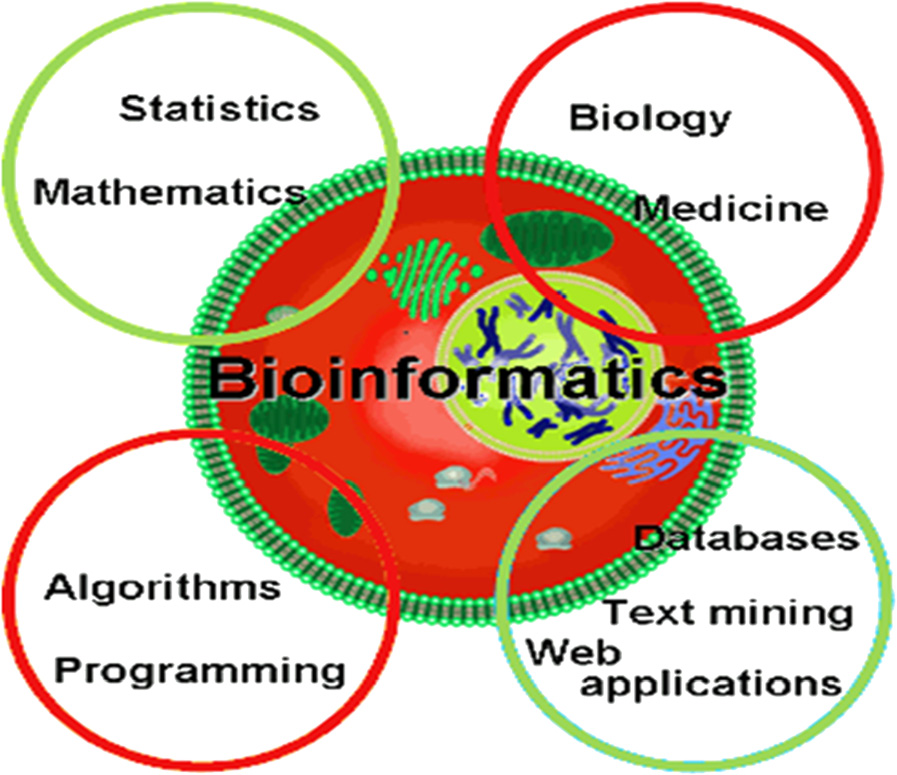
Four pillars of expertise in bioinformatics.

Genomics is a branch of science that deals with the common principles of genome infrastructure and function by DNA sequencing and genetic polymorphism analysis (Figures 5 and 6). The latter would provide an opportunity to develop a panel of PPPM-related algorithms and thereafter identify *pharmacotherapeutic* targets as the basis of developing tools of preventive gene-based therapy [10].

Recent advances in molecular biology have enabled a more detailed understanding of the impact of genetics as applicable to a full-term *clinical illness* and a *subclinical* stage of the disease, in particular. *Pharmacogenomics* is an allied portion of genomics and is thus a field of study to examine the impact of genetic variation on the response to medications. This approach is aimed at tailoring drug therapy at a dosage that is most appropriate for an individual patient, with the potential benefits of increasing the clinical efficacy and safety of medications [11]. Pharmacogenomics will guide therapeutic decisions and monitor the response to therapy.

The advances mentioned are converging with the movement towards consumer-driven health-care and patient empowerment. Whereas in the past medical testing was firmly under the control of medical practitioners, today’s genomic information is increasingly available outside the traditional medical settings. In the future, the primary role of healthcare professionals may be to interpret patients’ DTC genetic test results and advise them about appropriate follow-up.

### Transcriptomics

The *transcriptome* is regarded to be a set of all RNA molecules produced in one or a population of cells. The transcriptome can thus be seen as a precursor for the *proteome* (see below), that is, the entire set of proteins expressed by a *genome*.

The study of transcriptomics (see Figure 3), also referred to as expression profiling, examines the expression level of mRNAs in a given cell population, often using high-throughput techniques based on DNA microarray technology. Also, recent advances in RNAi screening and next-generation sequencing technologies enable a synergistic application of all of these genomic technologies to the discovery of *predictive* biomarkers [12,13].

### Proteomics

The fundamental role of proteomics (see Figure 3) belongs to the methods of the identification of individual proteins and *epitopes* within these proteins to be of value for *bioprediction*. The field of proteomics, or the comprehensive analysis and characterisation of all of the proteins and protein isoforms encoded by the human genome, may eventually have a great impact on PPPM [14].

**Figure 7:**
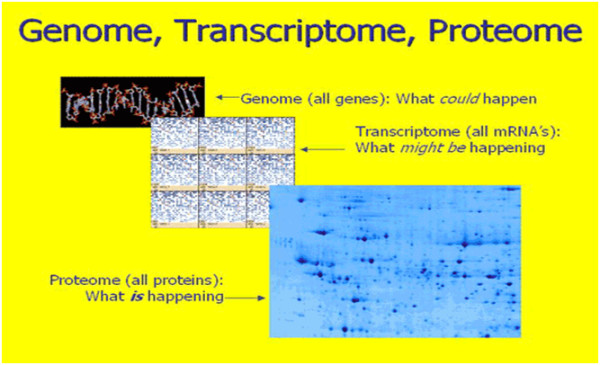
Genome, transcriptome, and proteome in a step-by-step assessment of possible risks and prediction of a latent/progressive disease.

It is hoped that recent advances in the understanding of the genetic aetiologies of common chronic diseases will improve pharmaceutical development. Thus, personalised medicine is in many ways simply an extension of traditional clinical medicine taking advantage of the cutting edge of genetic research [15,16]. In reality, proteomics *per se* is the continuation of *functional genomics* (see Figure 7) and, at the same time, the prologue to the following section—to metabolomics.

### Metabolomics

Metabolomics illustrates the functional state of the cell at the level of metabolism on a real-time basis, requiring the use of the term *metabolome*, uncustomary at first sight but reflecting a complex of all metabolic pathways in the cell at a given moment in time. Specifically, metabolomics (see Figure 3) is the ‘systematic study of the unique chemical fingerprints that specific cellular processes leave behind’, and the study of their small-molecule metabolite profiles.

One of the challenges for systems biology and functional genomics is to integrate proteomic, transcriptomic, and metabolomic information to give a more complete picture of living organisms (Figure 7*)*.

Fundamental science today as applicable to PPPM will thus demonstrate the following:

1. How the human genome has opened up a broad spectrum of predictive approaches for both simple and complex genetic diseases by the analysis of individual genes, SNPs, and haplotypes

2. How protein and RNA microarrays are providing new insight into the nature, course, and prognosis of certain ongoing diseases (e.g., cancer)

3. How autoantibodies (autoAbs) which now are known to be present years before the clinical onset of a number of autoimmune diseases (for instance, Type 1 diabetes (T1D), rheumatoid arthritis (RA), systemic lupus erythematosus (SLE), multiple sclerosis (MS), etc.) are being used as predictive markers to enter high-risk subjects into therapeutic intervention trials [17]. Thus, how is the whole data provided by metabolomics and, of course, genomics and proteomics to be comprehended?

### Bioinformatics as the essential stone in the overall PPPM concept

It is bioinformatics that serves to meet this goal by applying mathematical modelling techniques (see Figure 4). Bioinformatics deals with algorithms, databases and information systems, web technologies, artificial intelligence and soft computing, information and computation theory, structural biology and software engineering, etc. Bioinformatics generates new knowledge as well as the computational tools to create that knowledge.

**Figure 8:**
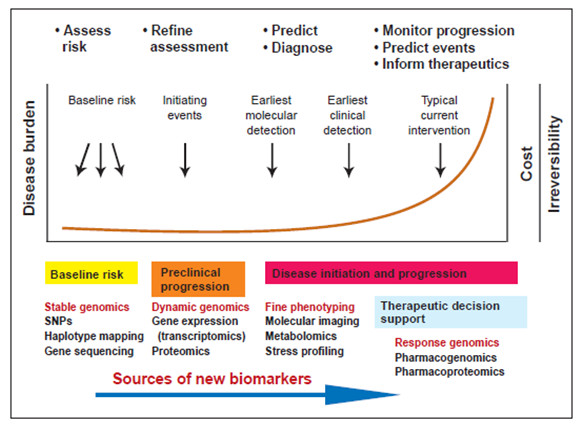
The multi-level graph delineating the relatedness of disease burden with costs and disease irreversibility along with the basic events in the course of a disease and instruments to be applicable for diagnosis and search for new biomarkers.

In the near future, *genotyping* and *phenotyping* results combined and consolidated under the aegis of having undergone computer-assisted processing will be used for the creation of unified information bases necessary for personal health biomonitoring, i.e., in terms of PPPM objectives, based on the principles of bioprediction and *bioprevention* through the stage of subclinical diagnostics (Figure 8) [18].

One idea of this medical model is the development of companion diagnostics, whereby molecular assays that measure the levels of proteins, genes, or specific mutations are used to provide a specific therapy for an individual’s condition by stratifying the disease status, selecting the proper medication, and tailoring dosages to the patient’s specific needs. Additionally, such methods might be used to assess the patient’s risk factors for a number of conditions and tailor individual preventive treatments such as nutritional immunology approaches. In the future, tissue-derived molecular information might be combined with an individual’s personal medical history, family history, and the data from imaging and other laboratory tests to develop more effective treatments for a wider variety of conditions [19].

An understanding and possibly a complete description of the factors underlying the burden of a disorder and later on of the disease will give policy makers, healthcare providers, and educators an opportunity to guide primary and secondary preventive initiatives at both individual and community levels.

### Aims, objectives, and tools of subclinical diagnostic armamentarium

PPPM uses diagnostic tests of newer generations, particularly genomic, proteomic, and metabolomic biomarkers, to individually determine the health conditions a person is predisposed to and to reveal the agents of the probable or the already existing pathological processes. The predictive branch is mainly designed to meet the interests of healthy individuals, its purpose being to determine whether susceptibility to a particular disease is increased or not. Preventive medicine is aimed at taking measures to avoid disease development rather than cure or treat it on manifestation. Finally, the model of personalised medicine proposes the customisation of healthcare, with all decisions and practices being tailored to the individual patient by the mutual integration of clinical information, stable and dynamic genomics, and molecular phenotyping through bioinformatics (Figure 1) [20,21].

**Figure 9:**
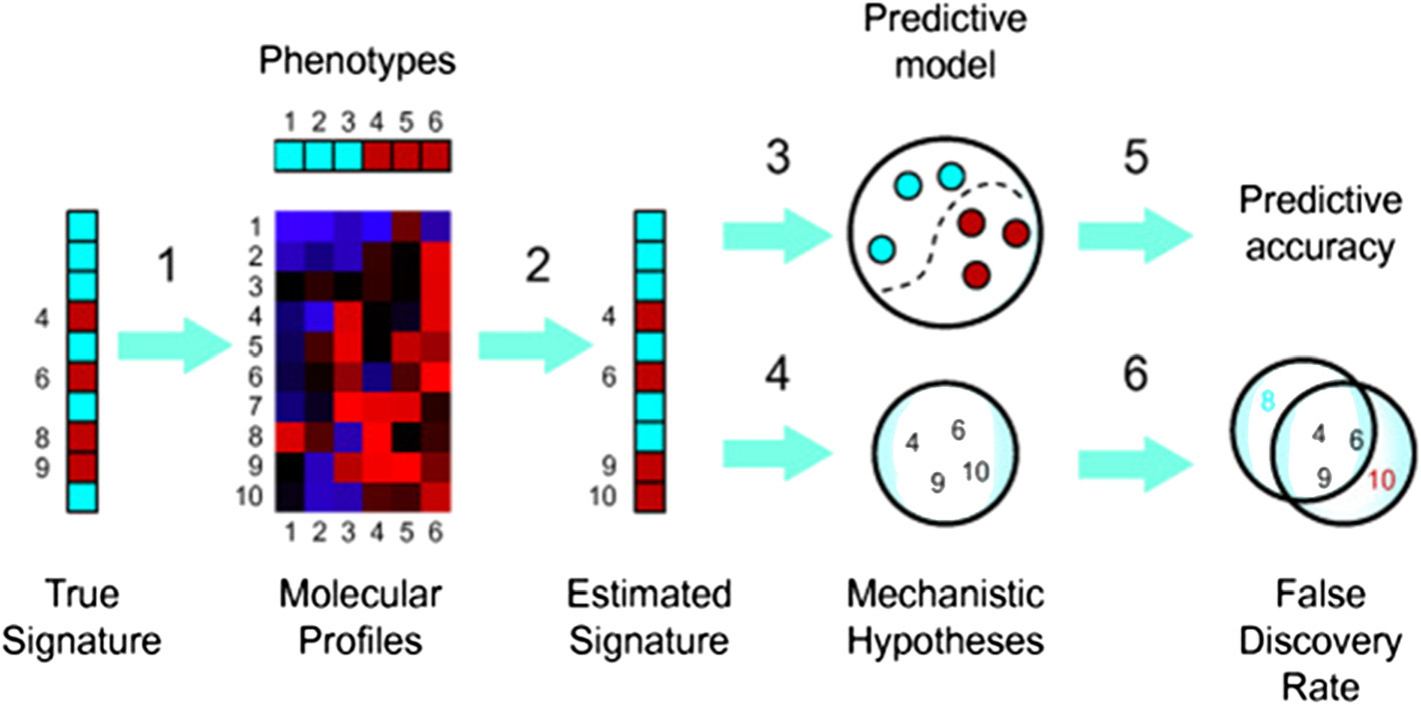
The fundamentals of BioChip methodology.

The future impacts of the application of a personalised medical approach can hardly be overestimated. For instance, it has become clear now that, in order to be successful, cancer treatments must be tailored to individual patients based on specific genetic drivers of tumour growth, and several preclinical platforms have already been developed for that purpose. Another example is drug resistance, which also proves dependent on gene–drug interactions affecting individual response to therapy. Generally, there is a multilevel infrastructure to demonstrate and to operate three levels desirable for providing optimal subclinical and clinical medical care services:

1. Determining genetic predisposition to a defined pathology to utilise updated protocols of genotyping*.* This step requires the use of such technologies as genetic polymorphism testing and DNA sequencing, as well as the analysis of information available from the genealogical tree, anamnesis morbi, and anamnesis vitae. Technologically, those goals can be accomplished by *BioChip* methodology (every disease has individual *fingerprints* and/or *molecular signatures*: changes in gene expression/transcription levels that are indicative of a nosology) (Figure 9) [22].

2. Individuals selected in the first stage undergo the second phase of the survey, which uses the target panel of *phenotypic biomarkers* (protocols of phenotyping);

3. Finally, the precise control of individual physiological responses to drug-based preventive measures is indispensible during the monitoring of dynamic shifts in the levels of biomarkers and *biopredictors.*

There has been much debate over securing the validity of PPPM-related genetic testing, possible risks, and benefits of PPPM, as well as some ethic issues [23]. Thus, we still have to answer a number of questions before we go too far down the PPPM-related path:

How can the quality and validity of genetic tests be ensured?

What are predictive medicine’s actual health benefits?

What are the risks and side effects associated with taking medicine before a person gets sick?

What are the psychological consequences of being told you are at risk of developing a certain disease?

How can third parties, such as employers and insurance providers, be prevented from using predictive medicine data in ways that negatively affect individuals?

### Subclinical diagnostics as applicable to particular diseases

**Figure 10:**
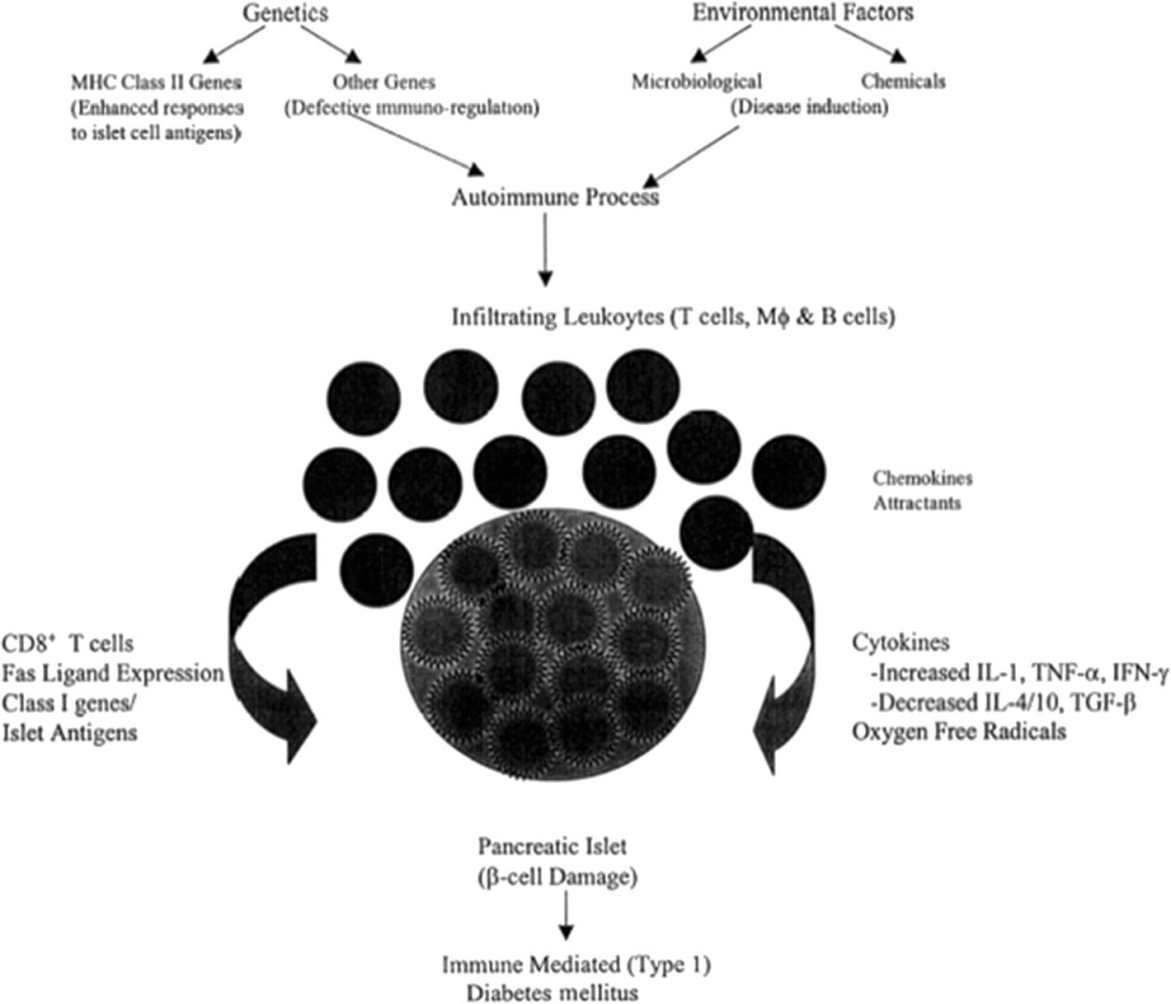
Aetiopathogenesis of T1D and critical events in dysregulation of immune system leading to β-cell loss and clinical manifestation.

There are two typical examples that best illustrate the specificity of the topic: T1D and MS. The basis of autoimmune diseases is a universal degenerative and inflammatory process, which comprises a number of stages, including the stage of subclinical pathology (see Figure 10).

Each stage is proved to be determined by a set of specific parameters, i.e.:

(a) Appearance of anti-islet autoAbs and an upsurge in the autoAbs titers in T1D, the most important biopredictive factor of T1D at the subclinical stage [24]

(b) Gene expression products of the key (e.g., functional transcripts) and anti-myelin autoAbs with proteolytic activity directed towards myelin antigens (Abs-proteases).

**Figure 11:**
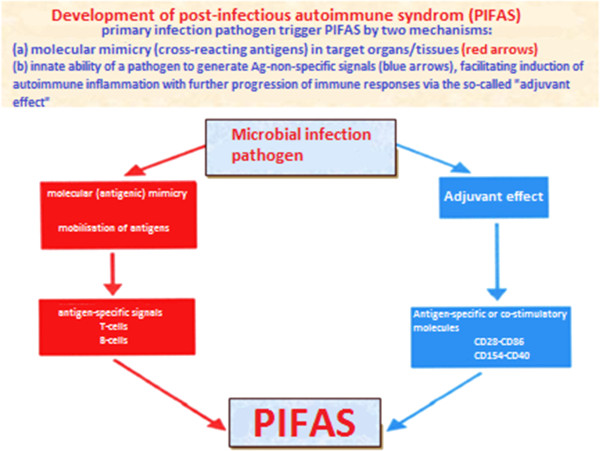
Two major mechanisms of PIFAS: molecular mimicry and adjuvant effect that are usually the common causes of most autoimmune diseases.

Measuring these allows the physician to assign proper treatment for persons at risks even at subclinical stage. The data set harvesting of genomics, proteomics, and metabolomics of an individual is an important approach to risk assessment for the relatives of persons with the diagnosed T1D or MS [25]. We have proposed a universal model of an autoimmune disease in a view of post-infectious autoimmune syndrome (PIFAS) (Figure 11), associated with the underlying disease, as a key factor to precede clinical manifestation and promote its chronisation [26].

**Figure 12:**
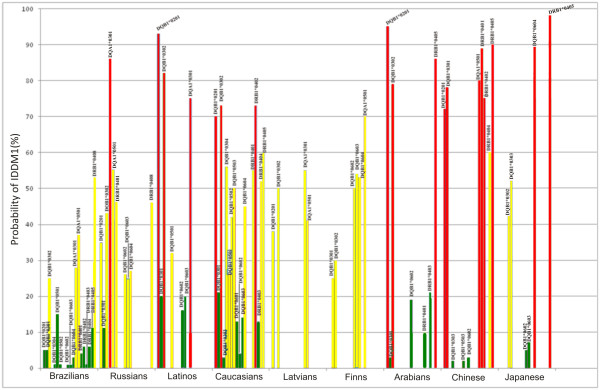
**The distribution of diabetogenic and protective potentials of HLA class II in different populations.***Green columns* refer to low risk (diabetoprotective); *yellow columns*, medium risk (moderately diabetogenic); and *red columns*, highly diabetogenic.

A spectrum of gene mutations to increase risks of T1D development is quite well determined. The most informative genetic markers of T1D from the list above are HLA-loci, particularly DR4, DR3, DQ, DR2, DR6, and DR7 (Figure 12), as their certain combinations promote the progression of PIFAS as a biopredictor of T1D-related clinical illness at a subclinical stage.

Concerning MS, the most important and informative gene combinations that have to date been associated with MS include 509 TGFB1, C DRB*18(3), CTLA4*G and 238TNF*B1, 308TNF*A2, and CTLA4*G. Such combinations support the formation of PIFAS-related signs at the subclinical stage, which are highly informative biopredictors to monitor a process of demyelination [27].

Proteomics, as applicable to the prediction of T1D, is no less important. T1D patients begin expressing autoAbs as early as 5–10 years before the clinical onset of the disease. Most of the data available indicate that this phenomenon also occurs in SLE, RA, Addison’s disease, celiac disease, and MS. Early evidence suggests that this phenomenon is also true to many of the 40–80 other autoimmune diseases identified so far [28,29]. The direct application of the aforementioned issues is just the combined gene*-* and phenotyping of persons at risks for biopredictors of both categories (genomics- and proteomics-related ones simultaneously) which would significantly raise the index of *predictivity* (up to 85%–90%), thus improving the selection of persons at risks for further drug-based therapeutic prevention [30].

### Preventive drug-based treatment as applicable to particular diseases

The strategy of drug-based therapeutic prevention in managing autoimmune diseases should include two critical points to make the subclinical diagnosis finalised and confirmed:

1. Quenching (arrest/blockage) autoagression and thus autoimmune chronic inflammation

2. Restoration of tissue- and organ-related morphofunctional architectonics.

The latter can successfully be achieved through the practical realisation of a number of strategies, particularly cell- and/or gene-based therapy, allogenic or xenogeneic transplantation, and stem cell technologies [31,32]. In addition, a principally new technology that holds promise in modern medicine is the application of Abs-proteases as novel tools for drug-based therapeutic prevention.

### Perspectives of PPPM as a tool in the global restructuring of the healthcare services

System approach to the formation of an innovative infrastructure regarding predictive and preventive algorithms is an ultimate approach that will contribute to the modernisation of the world healthcare services drastically. Our challenge is that the new guidelines should create the robust juristic and economic platforms for advanced medical services utilising the cost-effective models of risk assessments followed by tailored preventive treatments focused on the precursor stages of chronic diseases. Recently developed economical models clearly demonstrate the effectiveness of PPPM, if introduced as the integrative medical approach into the healthcare services [30].

Individuals to be under regular monitoring that helps to detect pathological shifts at subclinical stages of a disease have a higher life expectancy and are able-bodied up to 8–15 years more than those under traditional treatment. This means that the society saves more than US$20,000–40,000 per person annually [33].

However, above all, it is the people’s recognition of the responsibility for their own health and for the health of their children and their active involvement in preventive measures that can provide the strengthening of public health and the country’s biosafety through medical establishments. Therefore, with no government-directed and civil support, this national idea will remain without proper attention in spite of its perspective and social orientation [23]. This project requires the solution of several priorities.

First, it is the creation of a legal basis which would meet all society needs for the individual health to be protected—regulations of the state insurance as applicable to PPPM, financing channels of this sector including both budgetary and private sources, regulations of the doctor–patient relationships and, finally, the acquaintance with a new discipline to be fitted into the frame of the overall infrastructure of healthcare services [34]. Second, because of the novelty of this area, it is necessary not only to improve but also radically change the system of medical training, improving the level of their skills, expanding the technological spectrum of benefits for the population, and designing new approaches to build the academic schools of new generations.

There is a need to educate much more physicians and nurses in conducting hi-tech medical research. Moreover, there is a need to educate more clinicians to perform systematic reviews of previous research data. Only in this way are we able to close the gaps in our knowledge unveiling the outcome and benefits of PPPM.

Furthermore, there seems to be a need for more deep (in a stepwise manner) advising and mentoring students and younger colleagues of medical science and health services. The existing medical education would strongly need (apart of *graduate* and *post-graduate* levels) *pre-graduate* (higher school) level to disclose the mysteries of the evidence-based medicine. Thus, faculty advisors would have to advise a wide range of students and junior colleagues, from pre-graduate, through *predegree undergraduates* to *postdoctoral students* and *junior faculty* and *researchers.*

On the policy front, we must make sure that policies with respect to privacy, non-discrimination, and access to health insurance, all critical for any healthcare system, are aligned to maximise both the protections and the benefits to patients. The opportunity arises for unusual strategic partnerships between the government, the academy, and the commercial sectors to appear. The societal, ethical, and healthcare policy issues attendant to the anticipated changes will be profound. These changes must also be planned so that the barriers to the delivery of the benefits enabled by technical advances do not prevent their adoption.

## Conclusions

Meanwhile today, we are at the verge of global changes that illustrate the progress of medical healthcare. It becomes focused not on the therapy of an illness but rather on the protection of individual health. Also, PPPM would thus promise to sharply reverse the ever-escalating costs of healthcare-introducing diagnosis to stratify patients and disease, less expensive approaches to drug discovery, preventive medicine and wellness, and exponentially cost measurement technologies. PPPM also promises to improve patient outcomes and to empower both the patient and the physician [35]. We must accelerate this transformation by promoting the necessary scientific research and at the same time dealing with the societal challenges presented by PPPM. The healthcare industry, public policy sector, and consumer industries will of necessity be required to develop new and creative business models and products. There is a unique opportunity now to enable and accelerate a change by eliminating the key technical and societal (ethical, societal, policy, legal, economic, etc.) barriers that will prevent the full realisation of the revolution of PPPM. No doubt, the next generations will speak about the twenty-first century as a time when medicine became preventive and personified and its outcomes—predictive and guaranteed.

## Abbreviations

Abs: Antibodies; MS: Multiple sclerosis; PPPM: Predictive preventive and personalised medicine; RA: Rheumatoid arthritis; SLE: Systemic lupus erythematosus; T1D: Type 1 diabetes.

## Competing interests

The authors declare that they have no competing interests.

## Authors’ contributions

The authors contributed equally to the work. All authors read and approved the final manuscript.
